# The Divergent Pattern of SARS-CoV-2 Variant Predominance and Transmission Dynamics in the Brazilian Island of Ilhabela

**DOI:** 10.3390/v14071481

**Published:** 2022-07-05

**Authors:** Vincent Louis Viala, Svetoslav Nanev Slavov, Loyze Paola Oliveira de Lima, Alex Ranieri Jeronimo Lima, Gabriela Ribeiro, Antonio Jorge Martins, Bruna Petry, Cecilia Artico Banho, Claudia Renata dos Santos Barros, Cristina Tschorny Moncau, Debora Botequio Moretti, Debora Glenda Lima de La-Roque, Elaine Cristina Marqueze, Elisangela Chicaroni Mattos, Felipe Allan da Silva da Costa, Heidge Fukumasu, Jardelina de Souza Todao Bernardino, Jayme A. Souza-Neto, Jessika Cristina Chagas Lesbon, Lara Passos Kayanoki, Leandro Lombo Bernardo, Lívia Sacchetto, Luan Gaspar Clemente, Luiz Carlos Júnior Alcantara, Luiz Lehmann Coutinho, Beatriz de Carvalho Marques, Marta Giovanetti, Maurício Lacerda Nogueira, Mirele Daiana Poleti, Patricia Akemi Assato, Pedro De Queiroz Cattony Neto, Raquel de Lello Rocha Campos Cassano, Raul Machado Neto, Rejane Maria Tommasini Grotto, Ricardo Augusto Brassaloti, Simone Kashima, Dimas Tadeu Covas, Maria Carolina Elias, Sandra Coccuzzo Sampaio

**Affiliations:** 1 Butantan Institute, São Paulo 05503-900, SP, Brazil; loyze.lima@butantan.gov.br (L.P.O.d.L.); alex.lima@butantan.gov.br (A.R.J.L.); gabriela.rribeiro@butantan.gov.br (G.R.); antonio.martins@butantan.gov.br (A.J.M.); claudia.barros@butantan.gov.br (C.R.d.S.B.); debora.moretti@butantan.gov.br (D.B.M.); elaine.marqueze@butantan.gov.br (E.C.M.); jardelina.bernardino@butantan.gov.br (J.d.S.T.B.); pedro.cattony@butantan.gov.br (P.D.Q.C.N.); raul.machado@butantan.gov.br (R.M.N.); dimas.covas@butantan.gov.br (D.T.C.); 2 Blood Center of Ribeirão Preto, Ribeirão Preto Medical School, University of São Paulo, Ribeirão Preto 14049-900, SP, Brazil; svetoslav.slavov@hemocentro.fmrp.usp.br (S.N.S.); debora.laroque@usp.br (D.G.L.d.L.-R.); skashima@hemocentro.fmrp.usp.br (S.K.); 3 Centro de Genômica Funcional da ESALQ, University of São Paulo, Piracicaba 13418-900, SP, Brazil; bruna.petry@usp.br (B.P.); crismoncau@gmail.com (C.T.M.); luan.clemente@usp.br (L.G.C.); llcoutinho@usp.br (L.L.C.); raquel_lello@yahoo.com.br (R.d.L.R.C.C.); ricardo.brassaloti@usp.br (R.A.B.); 4 Medicine School of São José do Rio Preto (FAMERP), São José do Rio Preto 15090-000, SP, Brazil; ceci.abanho@gmail.com (C.A.B.); liviasacchetto@gmail.com (L.S.); bbiacarvalho@outlook.com (B.d.C.M.); mauricio.nogueira@edu.famerp.br (M.L.N.); 5 Department of Veterinary Medicine, School of Animal Science and Food Engineering, University of Sao Paulo, Pirassununga 13635-900, SP, Brazil; limattos@usp.br (E.C.M.); fukumasu@usp.br (H.F.); jessika.chagas@usp.br (J.C.C.L.); mirelep@usp.br (M.D.P.); 6 Department of Bioprocesses and Biotechnology, School of Agricultural Sciences, São Paulo State University (UNESP), Botucatu 18610-034, SP, Brazil; felipe.allan@unesp.br (F.A.d.S.d.C.); p.assato@unesp.br (P.A.A.); 7 School of Agricultural Sciences, São Paulo State University (UNESP), Botucatu 18610-034, SP, Brazil; jayme.souza-neto@unesp.br (J.A.S.-N.); rejane.grotto@unesp.br (R.M.T.G.); 8 Vigilância Epidemiológica de Ilhabela, Ilhabela 11630-000, SP, Brazil; ve.saude@ilhabela.sp.gov.br; 9 Hospital Mario Covas Junior, Ilhabela 11630-000, SP, Brazil; leandrobernardo50@gmail.com; 10 Instituto de Ciências Biológicas, Universidade Federal de Minas Gerais, Belo Horizonte 31270-901, MG, Brazil; alcantaraluiz42@gmail.com; 11 Instituto Oswaldo Cruz, FIOCRUZ, Rio de Janeiro 21040-360, RJ, Brazil; 12 Reference Laboratory of Flavivirus, Oswaldo Cruz Foundation, Rio de Janeiro 21040-360, RJ, Brazil; marta.giovanetti@ioc.fiocruz.br; 13 Department of Science and Technology for Humans and the Environment, University of Campus Bio-Medico di Roma, 00128 Rome, Italy; 14 Molecular Biology and Applied Biotechnology Laboratory, Clinical Hospital of the Botucatu Medical School, Botucatu 18610-034, SP, Brazil

**Keywords:** SARS-CoV-2, variants of concern (VOC), Brazil, molecular epidemiology

## Abstract

Our effort in SARS-CoV-2 genomic surveillance in Brazil has detected the Alpha Variant of Concern with a predominance higher than 75% in the population of Ilhabela island (São Paulo State) at a time when the Gamma VOC was already predominating the mainland raised concerns for closer surveillance on this island. Therefore, we intensified the surveillance for 24 weeks by generating data from 34% of local positive cases. Our data show that the patterns of VOC predominance dynamics and infection rates were in general distinct from the mainland. We report here the first known case of Alpha predominance in a Brazilian population, a delay greater than 3 months for the Gamma to dominate the previous variants compared to the mainland, and a faster dispersion rate of Gamma and Delta VOCs compared to the mainland. Phylogenetic analysis revealed the SARS-CoV-2 transmission dynamics in Ilhabela were characterized by multiple independent introduction events of Gamma and Delta, with a few events of Alpha introduction, two of them followed by community transmission. This study evidenced the peculiar behavior of SARS-CoV-2 variants in an isolated population and brought to light the importance of specific programs for SARS-CoV-2 genomic surveillance in isolated populations.

## 1. Introduction

The second wave of SARS-CoV-2 in Brazil was characterized by the rapid emergence and spread of the Gamma variant of concern (VOC) (P.1), which caused significant morbidity and mortality [1,2,3]. In the background of a fully dominant Gamma in Brazil, analysis of co-circulation of other SARS-CoV-2 lineages is important due to their complex interactions and different transmission and dissemination potential. To identify the circulating SARS-CoV-2 variants in the state of São Paulo, the Butantan Network for Pandemic Alert of SARS-CoV-2 Variant (Butantan Network) was established in March 2021, provided high throughput sequencing of SARS-CoV-2 of this Brazilian region. Of note, this network was responsible for the detection of the first cases of the Beta VOC (B.1.351) in Brazil [4], Lambda variant of interest (VOI) (C.37) in the State of São Paulo [5], and the initial steps of Delta VOC (B.1.617.2) introduction [2,6].

The state of São Paulo is the economic center of Brazil. The capital, also São Paulo, is the most populous city of South America and in the Southern Hemisphere, it has the most important airports and ports in Brazil. The geographic position of the State determines different landscapes from mountain ranges to a vast Atlantic coast with many archipelagos. The Butantan Network gives important information about the circulation of the SARS-CoV-2 of the whole state territory, including the insular region of Ilhabela (Atlantic coast of São Paulo State). It is reasonable to assume the island territories might represent a different profile of the SARS-CoV-2 variants due to specific characteristics like their isolation from the mainland, touristic hubs, human migrations, economic activities and lack of specialized healthcare facilities, all of which may determine the specific course of the SARS-CoV-2 pandemic. In this respect, during the routine SARS-CoV-2 genomic surveillance, we identified a high number of Alpha VOC samples in the island of Ilhabela, a profile which differs from continental São Paulo territory, where the Gamma VOC was already dominating the state. This finding required a more detailed investigation of the SARS-CoV-2 pandemic on this island, where we sequenced all available positive samples and observed peculiarities compared to the continental territory. Not only in the SARS-CoV-2 VOC composition but also in their replacement over time. Hereby, we report our observations on the SARS-CoV-2 pandemic in this particular island and discuss different aspects leading to this specific variant composition.

## 2. Materials and Methods

### 2.1. Sampling, Sample Preparation and Next-Generation Sequencing

From May 2021 to October 2021, nasopharyngeal swab samples were collected from Ilhabela inhabitants suspected to be infected with SARS-CoV-2 at the local Hospital: “Hospital Municipal De Ilhabela Governador Mario Covas Jr”. Viral RNA was isolated from 100 µL of nasopharyngeal swab suspension using the Extracta kit RNA viral (Loccus, Cotia, Brazil) in 96 well plate automated extractor Extracta (Loccus, Cotia, Brazil) following the manufacturer’s instructions. All samples were tested for SARS-CoV-2 by targeting viral RdRp, E, and *n* genes using the real-time PCR assay Gene FinderTM COVID19 Plus RealAmp kit (Osang Healthcare Co. Ltd., Anyang, Korea) and positive samples with Ct below 30 were selected for whole-genome sequencing. The RNA from positive samples was re-extracted from the original nasopharyngeal swab suspension for sequencing. The SARS-CoV-2 sequencing libraries were prepared using the COVIDSeq kit (Illumina Inc., San Diego, CA, USA), which amplifies the whole SARS-CoV-2 genome using the ARTIC v3 tilling PCR primer panel [7]. Pair-end libraries were sequenced on Illumina’s MiSeq (V2 kit, 2 × 150 cycles) or NextSeq 2000 (P2 kit, 2 × 100 cycles) platform.

### 2.2. SARS-CoV-2 Whole-Genome Assembly and Lineage Identification

Demultiplexed and adapter trimmed fastq files were recovered from the Illumina cloud BaseSpace Sequence Hub and submitted to another round of quality control analysis and trimming using FastQC v. 0.11.8 [8]. Reads were first trimmed for quality and ARTIC PCR primers using Trimmomatic v. 0.3.9 [9], then mapped to the Wuhan reference genome (Genbank refseq NC_045512.2) using BWA [10] and samtools v1.12 [11]. The assembly was refined for indels with two rounds of mapping using Pilon [12]. Finally, bcftools [13] was used for variant calling, and seqtk [14] (https://github.com/lh3/seqtk, accessed on 1 March 2020) for the creation of the consensus genomes. Positions covered by fewer than 10 reads (DP < 10) and bases of quality lower than 30 were considered a coverage gap and converted to Ns. Good quality genomes were considered for sequencing depth superior to 10 reads over more than 20 thousand nucleotides. The good quality genomes were then submitted to lineage identification with pangoLEARN version v1.2.101 (https://cov-lineages.org/lineages.html, accessed on 30 November 2021) [15].

### 2.3. Phylogenetic Analysis

The phylogenetic dataset was composed of 6744 genomes: 502 genomes from Ilhabela (genomes from this study sequenced between middle May and October 2021), 1982 genomes from the East region of the São Paulo State which is the closest mainland region from Ilhabela (genomes obtained from the genomic surveillance initiative Butantan Network), an additional 502 Alpha genomes from the SP State (also obtained from the Butantan Network) and 375 Alpha genomes from Brazil generated by other submitting laboratories downloaded from GISAID [16] to test the Alpha clustering hypothesis, and finally a global representative background dataset composed by 3383 genomes, also downloaded from GISAID. Only genomes > 29,000 bp and <1% of ambiguities were retrieved, low-quality genomes (>10% of ambiguous positions) were excluded. The phylogenetic analysis was performed using the Nextstrain v3.0.3 SARS-CoV-2 workflow [17]. The sequence alignment was performed using MAFFT v7.453 [18] with a subsequent realignment of misaligned stretches done in Aliview and removal of the genome tips to avoid regions of lower coverage [19]. Maximum Likelihood (ML) trees were estimated using IQ-TREE v1.6.12 [20] applying the maximum likelihood algorithm with statistical support of ultrafast bootstrap with 1000 replicates.

### 2.4. COVID-19 Incidence Dataset

The figures of new weekly COVID-19 cases from Ilhabela were obtained directly with the public agents of the Ilhabela health department (Secretaria da Saúde de Ilhabela). The number of new COVID-19 cases from the São Paulo State was downloaded the 1st December 2021 from the open access website (https://www.seade.gov.br/coronavirus/, accessed on 1 December 2021) of the São Paulo State public system of data analysis SEADE (Fundação Sistema Estadual de Análise de Dados). The new cases per 10,000 inhabitants was calculated based on the estimated population of each region available at the SEADE website (Ilhabela population = 33,821; East of São Paulo State population = 2,506,181; São Paulo State population = 45,003,221).

## 3. Results

### 3.1. SARS-CoV-2 VOC Predominance and Lineage Replacement on Ilhabela Island

**Figure 1:**
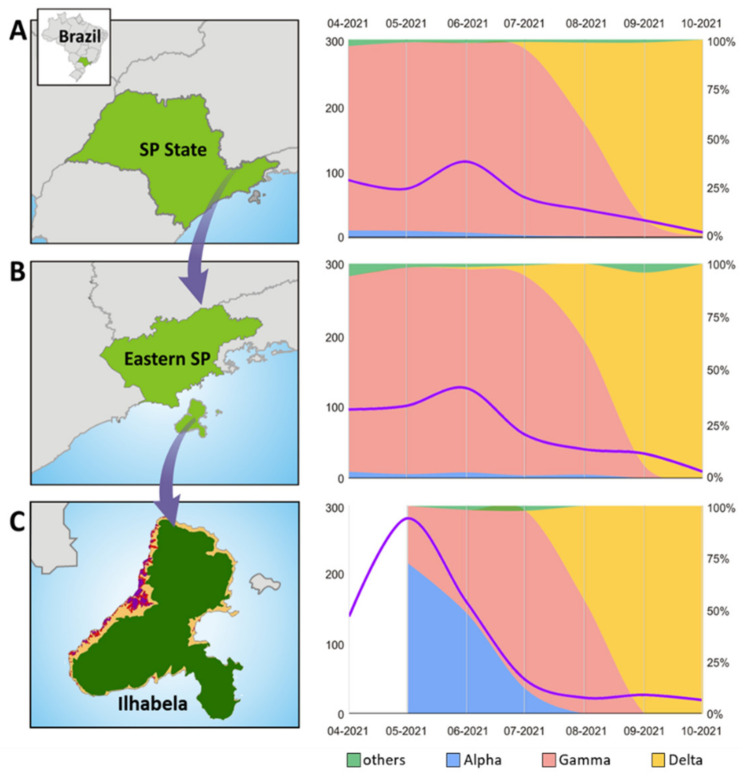
Comparison of the SARS-CoV-2 lineage predominance dynamics and record of new COVID-19 cases per 10 thousand inhabitants between São Paulo (SP) State (**A**), eastern SP (**B**) and Ilhabela island (**C**) from April to October 2021. The graph’s left axis indicates new cases per 10,000 inhabitants (purple line) and the right axis indicates the proportion of VOCs (Alpha VOC: blue area; Gamma VOC: light pink area; Delta VOC: yellow area; Other lineages: green area). The sampled areas are colored in light green in maps (**A**,**B**). The Ilhabela map (**C**) shows the protected forest area (dark green) and the unprotected area (yellow) with its denser urban spots (purple) (density map adapted from [21]).

Between middle May and October 2021, 516 positive samples for SARS-CoV-2 were sequenced, of which 502 (corresponding to 34% of all positive cases) were retained for lineage identification and reconstruction of the phylogenetic history of SARS-CoV-2 in Ilhabela. Based on the collection dates of the samples, we have analyzed the dynamics of VOC predominance along the period. The VOC dispersion dynamics on the island of Ilhabela (Figure 1) were compared to the data from the east region of São Paulo State (the nearest mainland region) and the São Paulo State as a whole.

It is clear that both new COVID-19 cases and the VOC replacement dynamics in Ilhabela are different from those observed in both mainland datasets (SP and eastern SP). From April to July, Ilhabela presented a much higher proportion of infections with its peak reaching around 280 new cases/10,000 inhabitants in May 2021 when compared to the mainland (less than half, around 130 new cases/10,000 inhabitants at its peak in July 2021). When comparing the VOC predominance, we can observe that the Alpha was nearly 78% prevalent during the first week of surveillance and then dropped gradually to extinction after 9 weeks of surveillance (averaging ~7% of decrease per week), when only the Gamma was detected. It is noteworthy that there was a huge delay in the timing of Gamma predominance on the island when compared to the mainland, as it became prevalent in the mainland 13 weeks before the island. Interestingly, the Gamma substitution rate toward domination (stable predominance >95%) in the island was distinct from the mainland which ranged from 61% to >95% in 9 weeks for the SP State, averaging 3.8% of increase per week, which is almost half of the Island speed (~7%). We also observed a higher speed of Delta substitution on the island when compared to the mainland. As we lack two weeks of genomic information on the island (epidemiological week 31 and 32), we are not sure exactly when the Delta has been introduced. Even considering it has entered the island during the first week of lacking data, we would observe a full dominance behavior of Delta over Gamma in only four weeks, thus averaging from 25% to 50% increase per week in contrast to an average of ~7% increase per week in the SP State and ~8% increase per week in eastern SP.

### 3.2. Phylogenetic Analysis

**Figure 2:**
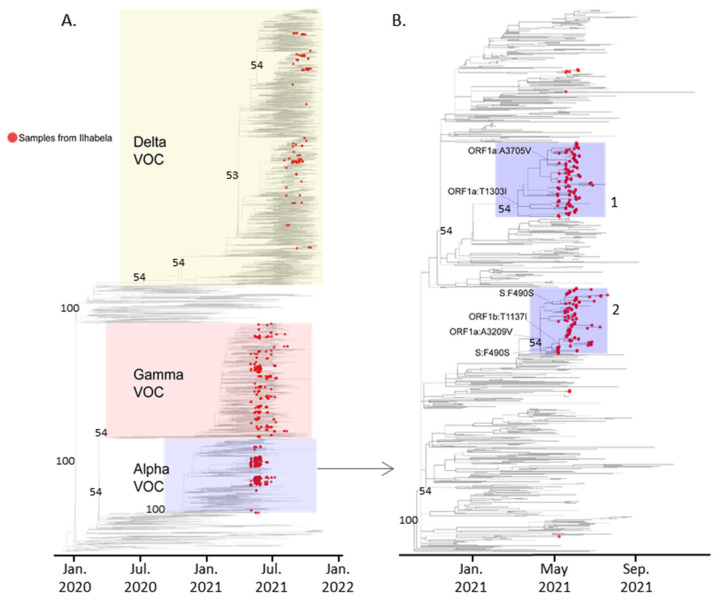
Maximum Likelihood time-tree of SARS-CoV-2 genomes of Ilhabela (red tips) inferred to a dataset with reference Alpha, Gamma and Delta VOC genomes obtained in Brazil and worldwide, downloaded from GISAID. Bootstrap values are presented for major branches. (**A**). Full dataset phylogeny with VOC clusters highlighted: Delta (light yellow background), Gamma (light pink background) and Alpha (light blue background). (**B**). Zoom in the Alpha branch showing the two monophyletic clustering of Ilhabela samples and their defining mutations.

The phylogenetic analysis performed over the SARS-CoV-2 genomes of Ilhabela, which were inferred to a dataset with reference Alpha, Gamma and Delta genomes obtained in Brazil and worldwide, indicate multiple independent introduction events of Gamma and Delta and few events of Alpha introduction on the island, two of them followed by community transmission (Figure 2).

The Alpha genomes collected from the Ilhabela population have separated mainly into two well-supported monophyletic clusters (Figure 2B), indicating two independent introductions of this VOC on the island, possibly followed by a sustained transmission within the island population. We have ruled out the possibility the Alpha clustering originated from an under-sampling bias by increasing the number of Alpha genomes with additional 466 Brazilian sequences. The persisting clustering, along with the fact they are almost completely composed of Ilhabela samples, reinforces that the Alpha has had a sustained transmission within the island. These two clusters of Alpha were characterized by the presence of defining non-synonymous mutations in the ORF1a (T1303I and A3209V respectively). One subclade of the monophyletic cluster 2 is defined by a non-synonymous mutation at the Spike protein (F490S). Despite the high rate of the Alpha on the island, it was fully substituted by Gamma by the end of July and the beginning of August. The phylogenetic analysis also demonstrated different small clusters which were randomly interspersed with other Brazilian Gamma strains, which differ from the Alpha, might be related to multiple introductions in the island (Figure 2A). The same was observed from Delta which was dominant since the beginning of September.

## 4. Discussion

During our routine SARS-CoV-2 genomic surveillance in the São Paulo State (Brazil), we observed an unexpectedly high predominance rate of the Alpha in the population of the Ilhabela island, located in the São Paulo State shore. Also, when comparing the proportion of new COVID-19 cases per inhabitants between Ilhabela and other continental regions, we observed higher rates of infections in Ilhabela. These surprising facts motivated closer genomic surveillance on this island population and our six-month effort allowed us to observe that this insular territory did not completely accompany the continent patterns of variant profile and infection rates. Our study brings insights into how the SARS-CoV-2 pandemic was shaped in an island territory despite the full dominance of Gamma in the continental territory of Brazil.

By the same period that Alpha was prevalent on the island, the Gamma was already dominant in Brazil [1,2,3] with limited dispersion of other VOCs and VOIs [4,5,22]. To our knowledge, there is no record of Alpha domination in Brazilian populations so far. Although some clusters of sustained transmission were attributed to the Alpha in the São Paulo State [22] its position and dissemination against the Gamma was insignificant. Different reasons could be discussed regarding the specific Alpha predominance in the island and its apparent sustained local transmission, as indicated in Figure 2 by the two large monophyletic clusters of local samples (two independent introductions followed by concomitant sustained transmission of both strains). On one hand, the island presents specific geographic and demographic characteristics such as a low but compact population (c.a. 30 thousand inhabitants) concentrated in 15% of the island territory (density of 414 inhab./km^2^) as 85% of the island is a protected forest (Ilhabela State Park). On the other hand, it has a single-entry point (ferry boat port) and the local government has implemented a negative RT-PCR test requirement or complete vaccination scheme for non-residents to enter the island between 26 March 2021 and 29 August 2021. Additionally, the local health agents have established a home monitoring team for the suspect and high-risk patients (elderly, people at social risk and with comorbidities) and implemented mass antigen testing in localities with large people circulation. These facts all together might have synergically contributed to promoting an enclosed environment to the Alpha transmission in the Island, ultimately leading to its predominance and late Gamma domination. Finally, the Ilhabela island is a major tourist hub in the São Paulo State and Brazil, which may have contributed at some point to the diversification of the SARS-CoV-2 profile. The same scenario could be attributed to explain the distinct rates of new infections (Supplementary Figure S1), but it is noteworthy that despite the high levels of new cases per inhabitant, the absolute number of cases (7428 until the writing of the manuscript) and the low levels of death (43 death) converged to one of the lowest lethality rates (0.6%) of SARS-CoV-2 in the State of São Paulo (Data from SEADE https://www.seade.gov.br/coronavirus/, accessed on 9 December 2021).

Our observations are similar to other studies which examine SARS-CoV-2 dynamics in insular territories. In Cyprus island it has been observed that the lineage dissemination and dynamics have been highly diversified possibly due to multiple introductions of different SARS-CoV-2 from Europe, the Americas and Asia which shaped the peculiar SARS-CoV-2 profile in the islands [23,24]. Considering the restriction measures, in large but insular territories like Ireland, it has been observed that social distancing and lockdowns have been related to a significant decrease in viral transmission [25], which in the Ilhabela case might have helped for the sustained transmission of Alpha compared to the continental territory and the delayed introduction of Gamma and Delta respectively.

In conclusion, we reinforce the importance of molecular genomic surveillance of SARS-CoV-2 lineage investigation and dissemination. With the implementation and expanding the use of daily SARS-CoV-2 molecular diagnosis and genomic sequencing, it was possible to track the real-time progression of the SARS-CoV-2 dissemination in the island which demonstrated variability compared to continental Brazil and is the only location of the State where the Alpha has been prevalent. Additionally, the variant substitution profile in the island was different compared to the continent. This study reinforces the importance of SARS-CoV-2 epidemiological surveillance in isolated population territories as they might present unexpected patterns of dissemination of variants becoming potential dissemination hotspots.

## Data Availability

The genomic sequences presented in this study are openly available in the GISAID public database (available online: https://www.gisaid.org/, accessed on 1 February 2022). The GISAID IDs and related metadata presented in this study are available in the Supplementary Materials.

## Supplementary Materials

The following supporting information can be downloaded at https://www.mdpi.com/article/10.3390/v14071481/s1, Table S1: Number of new cases and VOC surveillance data per epidemiological week for each region; Table S2: GISAID ids from the genomes used in the phylogenetic analysis; Figure S1: Comparison of the evolution of new COVID-19 cases per 10,000 inhabitants in 2021 between Ilhabela, São Paulo State and Eastern São Paulo State.
